# Supplementary material for: Continued need for non-pharmaceutical interventions after COVID-19 vaccination in long-term-care facilities
Source: Sci Rep. 2021 Sep 10;11:18093. doi: 10.1038/s41598-021-97612-w (PMC8433303; doi:10.1038/s41598-021-97612-w)
Supplement: Supplementary file 1 — Supplementary Information. [file 41598_2021_97612_MOESM1_ESM.docx]

**Supplemental Materials**

**Methods**

We developed a dynamic-network, agent-based model under a modified *Susceptible-Exposed-Infectious-Recovered* (SEIR) paradigm to simulate disease spread in LTCFs. The model was built in the R statistical environment ^1^. It uses a nested-loop structure which, while relatively computationally expensive, is highly intuitive and allows for individual-level record keeping and dynamics that scale up to stochastic population-level patterns. The model can be understood as two interacting components: the dynamic network model and the disease transmission model.

*The dynamic network model*

Upon initiation of a model simulation, three undirected contact matrices are generated: one for resident-resident contacts, one for worker-worker contacts, and one for resident-worker contacts. Together, these matrices describe a network of 100 residents and 51 workers. The initial condition for the resident network contains randomly generated contacts with mean degree of 2. The worker network is subdivided into 3 “shifts” to simulate more realistic coworker contact. Contacts in the worker network are also randomly generated, but contacts are exclusively between workers of the same shift. Probability of contact with a non-shiftmate is 0, whereas probability of contact with a shiftmate is 0.47, resulting in a mean degree of 8 in the worker network. Resident-worker contacts in the interaction matrix are generated randomly with probability based on the designated mean degree and node number of resident and worker matrices.

The network changes over the course of a simulation. Each timestep (day) begins with regeneration of each network, updating contacts randomly following the rules outlined above. No cohorting is assumed and the network is memory-less (i.e., past states do not influence future states). Workers may be removed from the network due to quarantine away from the facility (as described below in the disease transmission model section) by zeroing their contacts for that day, and their duties are assumed by one-day, non-repeating, non-infectious substitutes drawn from outside of the existing population of workers in the network. In this way, quarantined workers cannot spread disease but their absence does not impact features of the network other than decreasing the connectivity of would-be contacts of quarantined workers. Quarantining of residents is handled in a different manner (see below).

Residents and workers have contacts outside of the facility. Each resident has the same fixed daily probability of visitation (1/7, or an average of one outside contact per week). Worker degree of outside contact is heterogenous; each worker is assigned a level of outside contact drawn from a uniform distribution between 0 and 4.

*The disease transmission model*

Individuals can move between disease states (*Susceptible-Exposed-Infectious-Recovered*), which are explicitly designated at the individual level within the model, according to model parameters (Table S1). While disease transmission in the model occurs through daily application of an algorithm iterated across individuals rather than through a system of population-level mathematical equations, as would be in a traditional SEIR model, the model that we use can be reasonably visualized in the diagram in Figure S1. A key feature of the model designed to reflect COVID-19 disease dynamics is the split between the exposed-to-asymptomatic infectious and the exposed-to-presymptomatic infectious paths. The baseline probability of becoming presymptomatic was 60%, following CDC modeling recommendations. Immunity was considered to last for longer than the duration of the simulation (i.e., $\omega$ in Figure S1 is fixed at 0).

Individuals were quarantined if they developed symptomatic disease. Each symptomatic infection was assigned a score between 0 and 20 for disease severity. Since younger people tend to have lower rates of severe disease than older people, and since workers tend to be much younger than residents, worker disease severity for symptomatic cases was drawn from a normal distribution centered at 9 with a standard deviation of 2, while resident disease severity was drawn from a normal distribution centered at 15 with a standard deviation of 2. A cutoff value was established for the designation of severe disease at a severity score of 15. In this way, about half of symptomatic cases in residents become severe, while workers only rarely develop severe disease. Hospitalization cutoff value of severity was 17. Different death rates were defined for workers and residents and a daily death rate was derived considering length of infection for severe cases (see Table S1).

Individuals are quarantined upon development of symptoms. In contrast to quarantine of workers (as described above), quarantine of residents is “leaky”. For any contacts involving a symptomatic infectious resident, the infection rate parameter was reduced by a factor of 0.9.

Starting conditions assumed a completely naive population. Infectious spread begins when an individual is infected by a contact outside of the facility. Levels of outside contact described in the previous section determined the outside contact rate for each individual. Probability of an infection occurring though outside contacts was determined each day for each individual as a factor of the baseline infection rate, the level of outside contact for that individual, and the community prevalence of infectious COVID-19 (a value of 0.018 for these simulations, which reflects community prevalence in Salt Lake City, UT in early December, 2020).

One of our goals was to evaluate the impact of a vaccine with 95% efficacy as defined in clinical trials ^2^. This level of efficacy to prevent symptomatic disease could arise through a combination of prevention of infection and prevention of progression to symptomatic disease. We implemented these two potential modes of vaccine effectiveness in the model as distinct parameters that relate to each other through their joint contribution to vaccine efficacy: [1 – (1 – ψ)(1 – μ) = 0.95]. These factors modified probability of infection and probability of symptom development for vaccinated individuals, respectively. Residents had a 5% reduction in the effectiveness of these parameters. Vaccination coverage levels were determined at the start of each simulation and did not change across the duration of the simulated epidemic.

*The all-or-nothing case of vaccine action*

In our primary model and results, we assume that the vaccine is “leaky” in that it provides 95% immunity (through a combination of sterilizing and progression-blocking immunity) to 100% of those vaccinated. Another possibility is that the vaccine is “all-or-nothing” in that it would confer perfect immunity to only a portion of those vaccinated, while the remaining portion of those vaccinated would derive no immunity. To account for the possibility that vaccine efficacy is derived through an “all-or-nothing” action, we modified the model to simulate this scenario for a 95% efficacious vaccine as follows. Among those vaccinated, 95% will derive 100% immunity. Among those deriving 100% immunity, a random proportion will derive 100% sterilizing immunity ($\psi=1$ in the model), and the remaining proportion will derive 100% progression-blocking immunity ($\mu=1$ in the model). The results (Figures S2 and S3) show similar patterns to those found in the main results with “leaky” vaccine action.

*Transmission probability parameter value justification*

We assume that the “infectiousness” parameter, which defines the baseline probability of a contact between an infectious and a susceptible individual resulting in a successful exposure, is 1/9. Combined with the assumption that non-severe infections last 7 days, and that resident-resident contact rate is 2/day, we can use the formula $R0=\frac{Pinf\times Ncontacts}{\gamma}$, where *R0* is the basic reproductive number, *Pinf* is the infection probability, *Ncontacts* is the daily contact rate, and gamma is the rate of recovery from infectiousness, to yield *R0* =1.54. This reproductive number approximates the *R_effective_* found for SARS-CoV-2 in real populations during times of strict adherence to NPIs (CITE), and so our assumptions are realistic.

*Evaluating patterns of infection and disease over a longer timecourse*

Though our study focuses on relatively short-term (100 day) epidemiology of SARS-CoV-2 in LTCF settings, we also conducted simulations over a longer span of time to determine if long-term effects would be different. We found that patterns of infection and disease were similar in these longer simulations. Infections, disease, and deaths increase in longer simulations relative to their shorter counterparts, but the effects of adherence to NPIs remain very similar (Figures S4 and S5). Importantly, the impact of vaccinating healthcare workers is substantially lower in these 500 day simulations than in 100 day simulations. This analysis therefore further exaggerates the suggestion that prioritizing vaccination of residents over healthcare workers may be favored under certain conditions, as discussed in the main text.

*Calculating Vaccine Effectiveness*

We calculated vaccine effectiveness with respect to symptomatic disease from the simulation outputs as 1 minus the prevalence of symptomatic disease in the vaccinated versus the unvaccinated population. In the simulated data, vaccine effectiveness with respect to symptomatic disease shows a minor decline with increasing ψ values (Figure S6). Vaccine effectiveness with respect to disease was found to be slightly higher in Scenario 1 (strict adherence to NPIs) than in Scenarios 2 and 3 (waning and weak NPI adherence, respectively; Figure 3). Vaccine effectiveness as measured in the model output data does not reach 95%, the modeled vaccine efficacy. This is likely to be partially due to stochasticity; when a simulated epidemic does not spread substantially in the population because of stochasic unpredictability of contacts, then numbers of infected are low and the calculation for vaccine effectiveness is based on a small number of infections.

*Visualizations*

Figure 2 depicts heatmaps that compare effects of vaccine coverage levels on deaths across three scenarios. These heatmaps were created by fitting a second-degree loess smoothing function with a span value of 0.5 to the data and interpolating to all values within the data range, then producing plots with the levelplot function from the lattice package in R ^3^.

**References**

1. R Core Team. R: A language and environment for statistical computing. (2017).

2. Polack, F. P. *et al.* Safety and Efficacy of the BNT162b2 mRNA Covid-19 Vaccine. *N Engl J Med* 1–13 (2020). doi:10.1056/NEJMoa2034577

3. Sarkar, D. *Lattice: Multivariate Data Visualization with R*. (Springer, 2008).

Table S1. Parameter values.


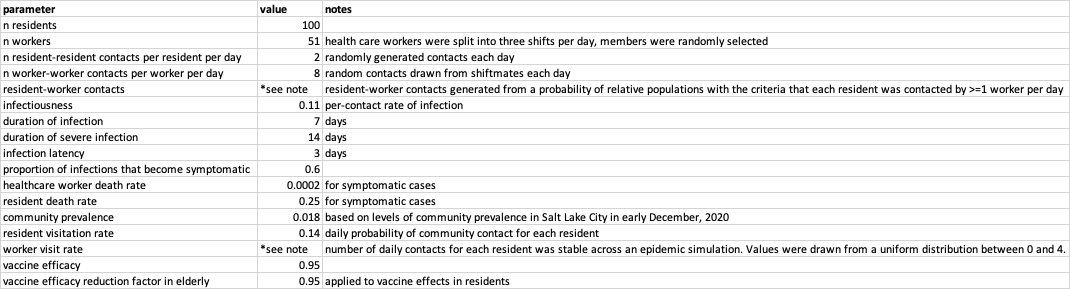


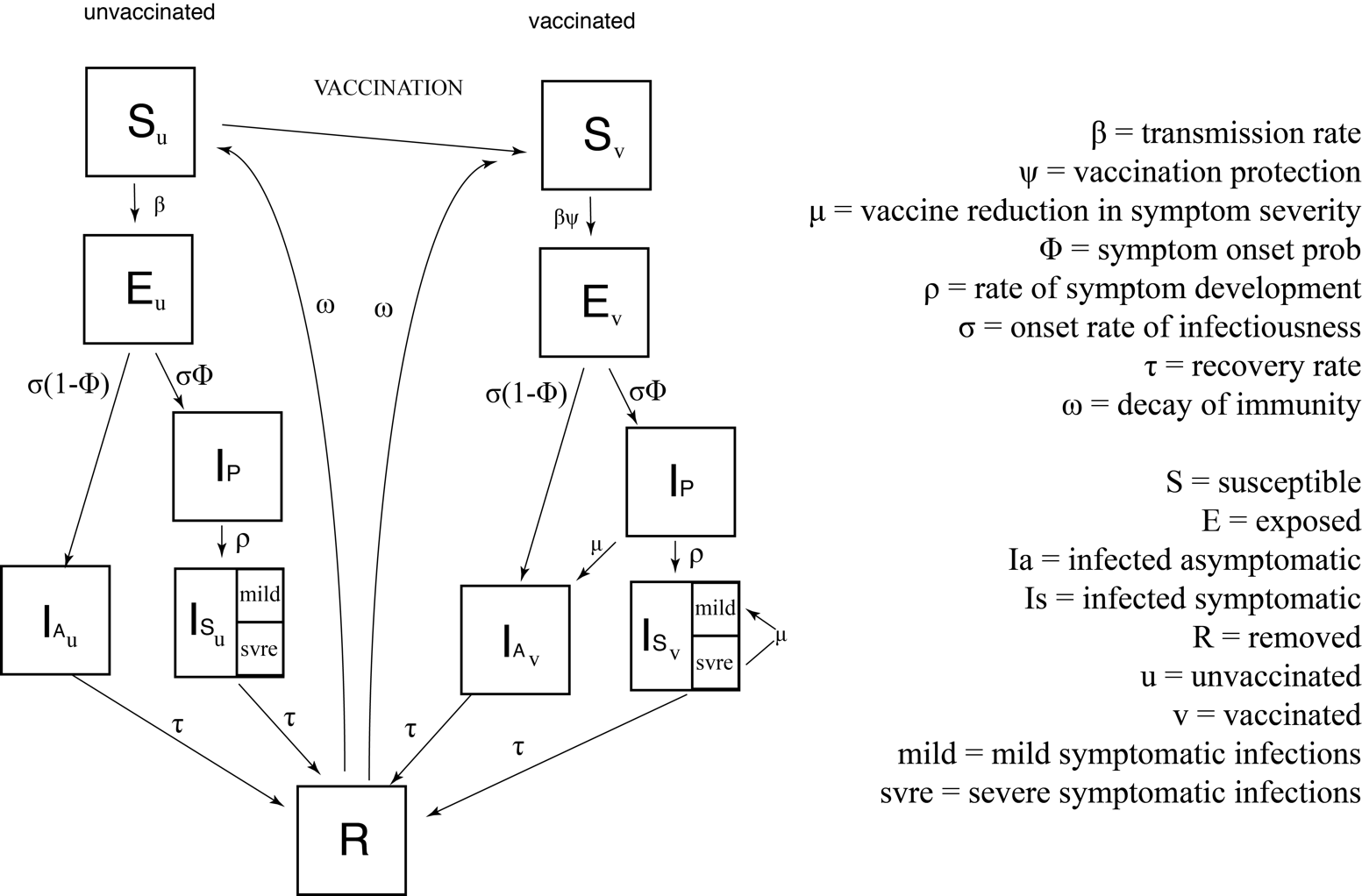


Figure S1. Model diagram. Individuals move between S, E, Ia, Ip, Is, and R compartments according to the rate parameters, applied as daily probabilities at each time step. Vaccination can affect dynamics of the system through ψ and μ.


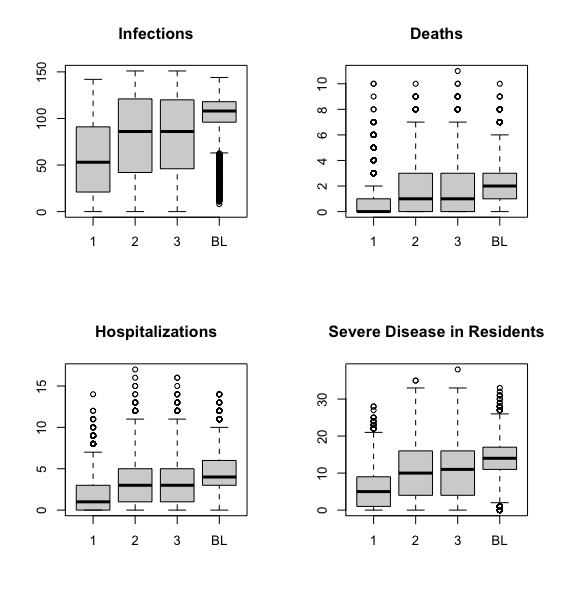


Figure S2. Disease outcomes by scenario for the all-or-nothing case of vaccine action. Comparison of total infections, severe infections in residents, hospitalizations, and deaths over 100 days in simulated populations of 100 residents and 51 healthcare workers at long-term care facilities. Scenarios 1, 2, and 3 paired vaccination with strong, gradually reduced, and weak adherence to NPIs, respectively. Scenario BL (baseline) included no vaccination but strong adherence to NPIs. As in the “leaky” vaccine action case, in the all-or-nothing case, reduction in infection and disease burden was weaker under relaxing or weak NPI adherence when compared to the strong NPI adherence scenario. Boxplots show median, 1^st^ and 3^rd^ quartile, and whiskers extend to 1.5 times the interquartile range. Statistical outliers included as open circles.


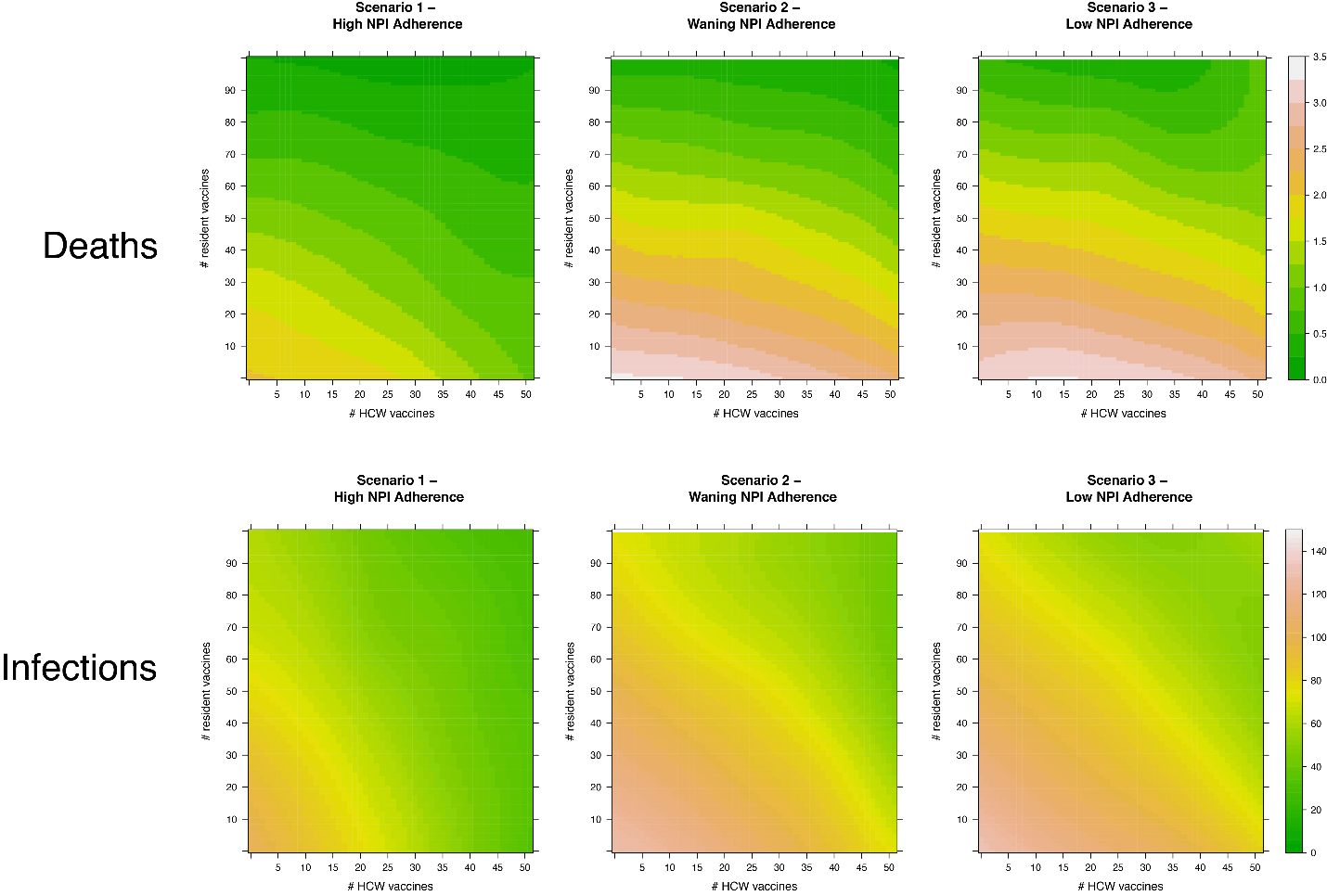


Figure S3. Impact of vaccine coverage on COVID-19 deaths (top row) and infections (bottom row) in the case of an all-or-nothing vaccine. Heatmaps of the impact of vaccine coverage on COVID-19 deaths (top) and infections (bottom) in simulated long-term care facility populations of 51 healthcare workers and 100 residents for three scenarios with different NPI adherence. Warmer colors indicate more deaths/infections. As in the “leaky” case, per vaccine, vaccinating healthcare workers prevents more deaths when NPI adherence is high (Scenario 1, left), but that impact declines substantially when NPI adherence wanes (Scenario 2, middle) or is low (Scenario 3, right).


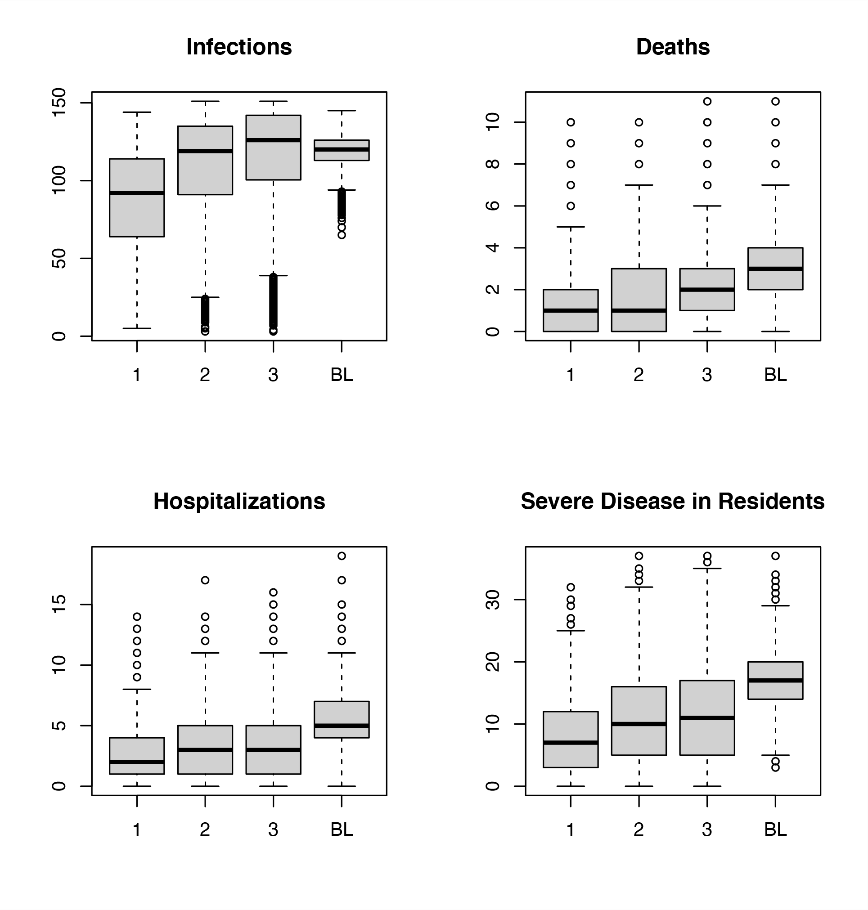


Figure S4. Disease outcomes by scenario for 500 day simulations. All other parameter values were the same as in the 100 day simulations (See Figure 1 in the main text). Comparison of total infections, severe infections in residents, hospitalizations, and deaths over 100 days in simulated populations of 100 residents and 51 healthcare workers at long-term care facilities. Scenarios 1, 2, and 3 paired vaccination with strong, gradually reduced, and weak adherence to NPIs, respectively. Scenario BL (baseline) included no vaccination but strong adherence to NPIs. As in the 100-day simulations, in the 500 day simulations show that reduction in disease burden was weaker under relaxing or weak NPI adherence when compared to the strong NPI adherence scenario. In these longer simulations, Scenarios 2 and 3 showed marginal reduction in total infections compared to Baseline. Boxplots show median, 1^st^ and 3^rd^ quartile, and whiskers extend to 1.5 times the interquartile range. Statistical outliers included as open circles.


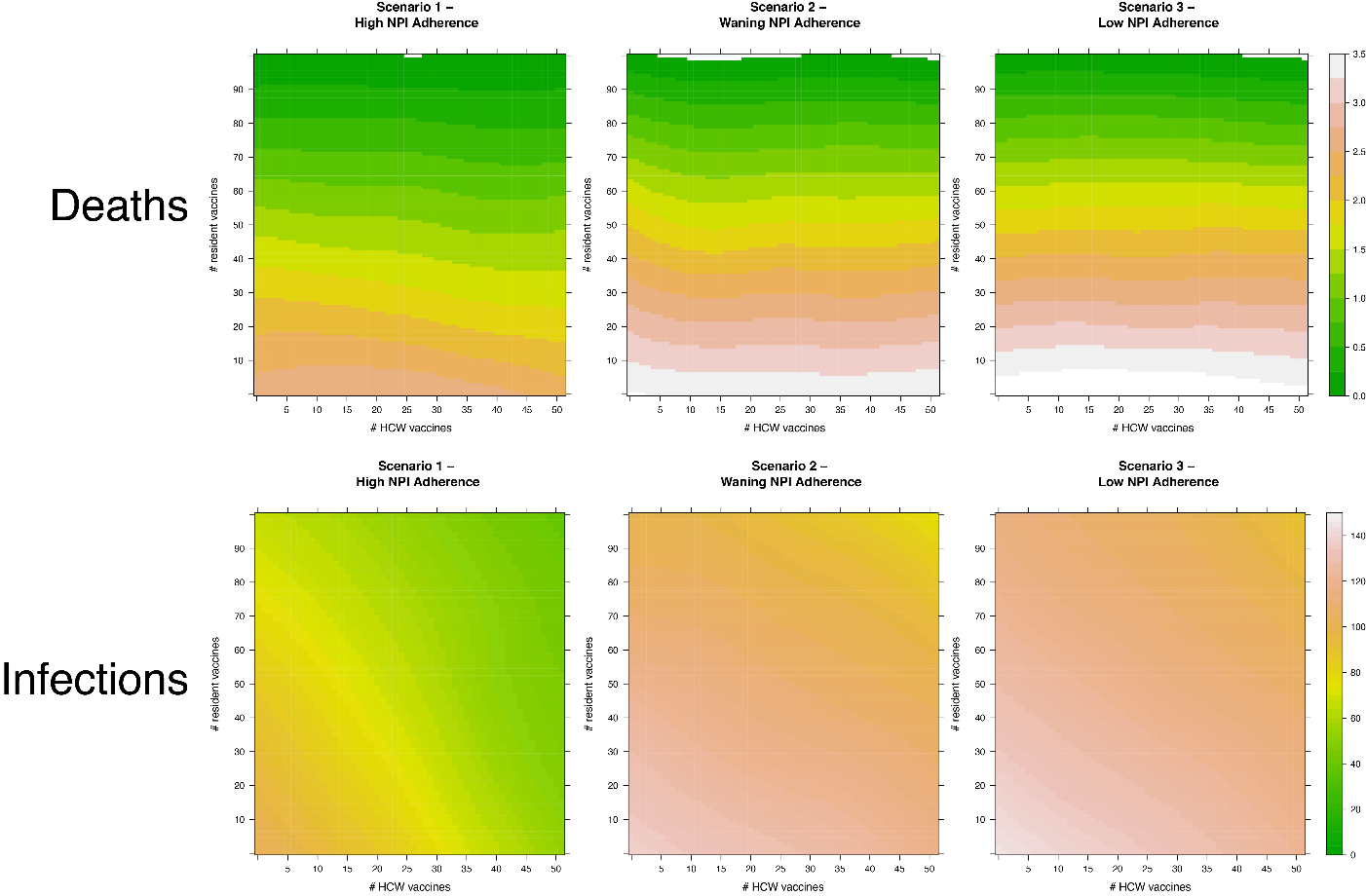


Figure S5. Impact of vaccine coverage on COVID-19 deaths (top row) and infections (bottom row) in 500 day simulations. Heatmaps of the impact of vaccine coverage on COVID-19 deaths (top) and infections (bottom) in simulated long-term care facility populations of 51 healthcare workers and 100 residents for three scenarios with different NPI adherence. Warmer colors indicate more deaths/infections. As in the 100 day simulations, per vaccine, vaccinating healthcare workers prevents more deaths when NPI adherence is high (Scenario 1, left), but that impact declines substantially when NPI adherence wanes (Scenario 2, middle) or is low (Scenario 3, right). The effect of vaccinating healthcare workers on mortality is substantially reduced in these 500 day simulations when compared to 100 day simulations.

Figure S6. Measured vaccine effectiveness was high and showed a mild contingency on the degree to which the vaccine conferred sterilizing immunity (ψ) and adherence to NPIs (Scenarios) in the simulations. The plot shows curves of GAM models fit to the simulated data (black circles) across all vaccine coverage levels with a 95% efficacious vaccine and illustrates relationships between ψ and vaccine effectiveness with respect to symptomatic disease. Scenario 1, with high NPI adherence, showed higher overall effectiveness than scenarios 2 and 3, with waning and low adherence, respectively.
